# Modulating the Afterglow Time of Mn^2+^ Doped Metal Halides and Applications in Advanced Optical Information Encryption

**DOI:** 10.3390/nano15131002

**Published:** 2025-06-28

**Authors:** Yu-Lin Hu, Yi-Lin Zhu, Shi-Ying Gu, Jia-Qing Xu, Zhi-Xing Gan, Chuan-Guo Shi

**Affiliations:** 1 School of Chemistry and Chemical Engineering, Nantong University, Nantong 226019, China; 2 School of Public Health, Nantong University, Nantong 226019, China; 3 School of Intelligent Manufacturing and Electronic Engineering, Wenzhou University of Technology, Wenzhou 325035, China; 4 School of Computer and Electronic Information/School of Artificial Intelligence, Nanjing Normal University, Nanjing 210023, China

**Keywords:** afterglow, photoluminescence, optical information storage

## Abstract

Mn^2+^ doped metal halide that can be grown by a facile solution reaction is a promising low-cost afterglow material. However, the afterglow mechanism is still elusive. Using a facile method to modulate afterglow time is still to be explored. In this work, we reveal that the afterglow of Cs_2_Na_0.2_Ag_0.8_InCl_6_:y%Mn can be significantly modulated by Mn^2+^ concentration. We propose that replacing Ag^+^ with Mn^2+^ leads to the appearance of interstitial Ag^+^, which temporally store the photogenerated electrons (${Ag}^{+}+e^{-}\to Ag$). After the removal of excitation, the gradual recombination between residual holes and stored electrons [$h^{+}+\left({Ag}^{+}+e^{-}\right)\to h\nu +{Ag}^{+}$] explains the afterglow. However, excessive Mn^2+^ doping at interstitial sites does not bring about more interstitial Ag^+^ but instead introduces nonradiative traps. Therefore, as the Mn^2+^ concentration increases, the afterglow time increases from 350 s to 530 s and then decreases to 230 s, reaching a maximum at y = 40. Thus, a dynamic optical information storage and encryption application is demonstrated based on the modulated afterglow time.

## 1. Introduction

Afterglow phosphors have exhibited a wide range of applications in the fields of advanced anti-counterfeiting, optical information storage, optical imaging, biological detection, lighting etc. Very recently, Lv et al. reported an appealing deep-trap ultraviolet storage phosphor, ScBO_3_:Bi^3+^, which exhibited an ultra-narrowband light emission centered at 299 nm [1]. Based on the unique spectral features and trap distribution, controllable optical information read-out is demonstrated via external light or heat manipulation, highlighting the great potential for advanced optical storage application in bright environments. Sheng et al. proposed the co-multiplexing spectral and temporal dimensions based on photoluminescence (PL) and corresponding afterglow at four different wavelengths [2]. Each emission color shows four possible emissive modes (4^4^), representing the maximum encoding capacity of 8 bits at each pixel. Furthermore, photonic synapses that emulate the human visual system are attracting research interest [3]. Afterglow also shows application potential in photonic synapses [4,5]. Lu et al. proposed an optically stimulated optical-response artificial synapse based on the afterglow material (SrAl_2_O_4_:Eu^2+^, Dy^3+^) [4]. The output intensity gradually decayed to initial state, suggesting a photo-memory characteristic, which was used to imitate the typical excitatory postsynaptic behavior of a biological synapse. Zhang et al. developed a frosted luminescent solar concentrator (FLSC) by incorporating the Si_5.9_Al_0.1_O_0.1_N_7.9_:Eu^2+^ phosphor and the afterglow SrAl_2_O_4_:Eu^2+^, Dy^3+^ phosphor into a thiol-ene polymer [6]. In addition to photovoltaic modules, the FLSC also acts as a zero-energy nightscape lighting device due to the persistent luminescence [6].

Until now, several afterglow phosphors have already been commercialized, such as white emitting (Li,Na)_8_Al_6_Si_6_O_24_(Cl,S)_2_:Ti and green emitting SrAl_2_O_4_:Eu^2+^. Although the oxides are highly stable, their large lattice formation energy suggests a high synthesis temperature and huge energy consumption. Besides, the available emission wavelengths are also relatively limited. Thus, it is still necessary to develop more afterglow phosphors. The ability of carbon dots to emit afterglow has attracted research interest. However, their afterglow efficiency and lifetime need further improvement [7]. Owing to the high absorption coefficient, tunable bandgap, defect-tolerant nature, high photoluminescence quantum yield (PLQY), high charge-carrier mobility, and simple solution synthesis, inorganic lead halide perovskites have attracted attention in the field of afterglow [8,9,10,11]. Zhang et al. replaced Pb^2+^ with appropriate lanthanide ions (Ln^3+^) in CsPbBr_3_ nanocrystals, which were embedded inside an amorphous transparent medium [12]. The CsPbBr_3_:Ln^3+^ nanocrystals emitted a green afterglow lasting 1800 s. However, the instability and the toxicity of lead ions have limited their practical applications.

During the past several years, lead-free metal halides such as Cs_2_AgInX_6_, Cs_2_AgBiX_6_, and Cs_2_NaInCl_6_ and their low dimensional derivatives have been widely investigated due to their lower toxicity and better stability compared to CsPbX_3_ [13,14,15,16,17,18]. Among them, Cs_2_AgInCl_6_ is one of the most noticeable metal halides due to its direct bandgap. Zheng et al. synthesized Cs_2_Na_x_Ag_1-x_InCl_6_:Mn via a facile hydrothermal reaction at 180 °C, which exhibited a red afterglow [19]. Yang et al. reported the synthesis of Mn^2+^/Yb^3+^/Er^3+^ tri-doped Cs_2_Ag_0.8_Na_0.2_InCl_6_, which exhibited both visible and NIR afterglow due to the release of long-lived trapped electrons to Mn^2+^ and Er^3+^ ions [20]. Multi-modal emissions of the tri-doped metal halides enable a five-level anti-counterfeiting strategy. Therefore, the Mn^2+^ doped metal halides provide a promising platform for the development of afterglow phosphors. However, the nature of the long-lived trap (LLT) is still elusive. A facile method to modulate their afterglow time is still to be explored.

In this work, Cs_2_Na_0.2_Ag_0.8_InCl_6_:y%Mn crystals were synthesized by a solvothermal method, which showed afterglow emission at about 600 nm. As the y value increased from 20 to 60, the afterglow time increased first and then decreased, with a maximum of 530 s at y = 40. To understand the Mn^2+^ concentration dependent afterglow time, steady-state photoluminescence (PL) spectra and time resolved PL, the ultraviolet–visible (UV–vis) diffuse reflectance spectra of Cs_2_Na_0.2_Ag_0.8_InCl_6_:y%Mn crystals were measured and analyzed. Their crystal structures and band structures were analyzed based on X-ray diffraction (XRD) patterns, high resolution transmission electron microscopy (HRTEM) images, and ultraviolet photoelectron spectra (UPS). On the one hand, the substitution of Ag^+^ by Mn^2+^ leads to the appearance of interstitial Ag^+^, which temporally store the photogenerated electrons due to the photoreduction reaction. The release of carriers results in afterglow. On the other hand, the Mn^2+^ doping introduces nonradiative traps near the band edges, leading to the monotonic descending of both the PL and afterglow intensity with increasing Mn^2+^ concentration. In particular, excessive Mn^2+^ doping at interstitial sites does not bring about more interstitial Ag^+^ but rather introduces nonradiative traps. Thus, the afterglow time first increases then decreases as the Mn^2+^ doping concentration increases. Based on the modulated afterglow time, an optical information storage and encryption application is proposed.

## 2. Materials and Methods

### 2.1. Materials

Cesium chloride (CsCl, 99.99%), silver chloride (AgCl, 99.5%), indium chloride (InCl_3_, 99.99%), and manganese chloride (MnCl_2_, 99%) were obtained from Shanghai Aladdin Ltd. (Shanghai, China) Ethylenediaminetetraacetic acid disodium salt (EDTA-2Na·2H_2_O, 99%), hydrochloric acid (HCl, 12 M), polymethyl methacrylate (PMMA, M.W. 35,000), and N, N-dimethylformamide (DMF, 99.7%) were purchased from Sinopharm Chemical Reagent Co, Ltd. (Shanghai, China). All chemicals were used as received without further purification.

### 2.2. Synthesis of Cs_2_Na_0.2_Ag_0.8_InCl_6_:y%Mn

To synthesize Cs_2_Na_0.2_Ag_0.8_InCl_6_:y%Mn, 2 mmol CsCl, 0.8 mmol AgCl, 1 mmol InCl_3_, y mmol MnCl_2_, and 0.1 mmol EDTA-2Na·2H_2_O were loaded in a 25 mL polytetrafluoroethylene vessel, followed by the addition of 12 mL of concentrated hydrochloric acid. The vessel was sealed in a stainless-steel autoclave, which was then kept in an oven at 180 °C for 12 h. The crystallization was started by slow-cooling to 30 °C at a rate of ~3 °C/h. The bottom crystals were rinsed three times with isopropanol and then dried on filter paper at room temperature. The crystals with Mn^2+^ doping were lightly crushed and sieved with 50 mesh to obtain powder crystals and then stored in hooded vials for further characterizations.

### 2.3. Structural and Optical Characterizations

Powder X-ray diffraction (XRD) patterns were collected using an X-ray diffractometer (Bruker D8, Billerica, MA, USA) coupled with Cu-K radiation. The scanning rate was set at 8°/min with a step size of 0.02°. High resolution transmission electron microscopy (HRTEM) images were obtained using a transmission electron microscope (TEM, JEOL, JEM-2100PLUS, Tokyo, Japan). In the HRTEM measurements, we precisely adjusted the electron beam direction at 0.1°/step to align the crystal plane axis. Ultraviolet–visible (UV–vis) diffuse reflectance spectra in the range of 200–800 nm were recorded by a spectrophotometer (Cary 5000, Varian, Palo Alto, CA, USA). X-ray and ultraviolet photoelectron spectroscopy were measured by Thermo ESCALAB 250Xi (Sydney, Australia) with an Al Ka source. To determine VBM accurately, a linear tangent was used to fit the steepest part of the leading edge of UPS, where the intensity starts to increase. Then, this tangent was extrapolated to intersect the baseline. The BE at this intersection is the VBM. The measurements of the PL and afterglow fluorescence were conducted in a spectrofluorometer (FLS-1000 spectrometer, Edinburgh Instrument, Edinburgh, Scotland). The excitation wavelength was set at 365 nm. For measurements of the PL lifetime, a μs flash lamp was used as the excitation source. Afterglow time was obtained with a phosphorophotometer (Pr-305, HangZhou ZheJiang University Sensing Instruments Co., Ltd., Hangzhou, China). All of the afterglow measurements were conducted in ambient air. The afterglow time is defined as the period between the removal of UV excitation and when the afterglow intensity decreases to background noise level.

## 3. Results and Discussion

**Figure 1:**
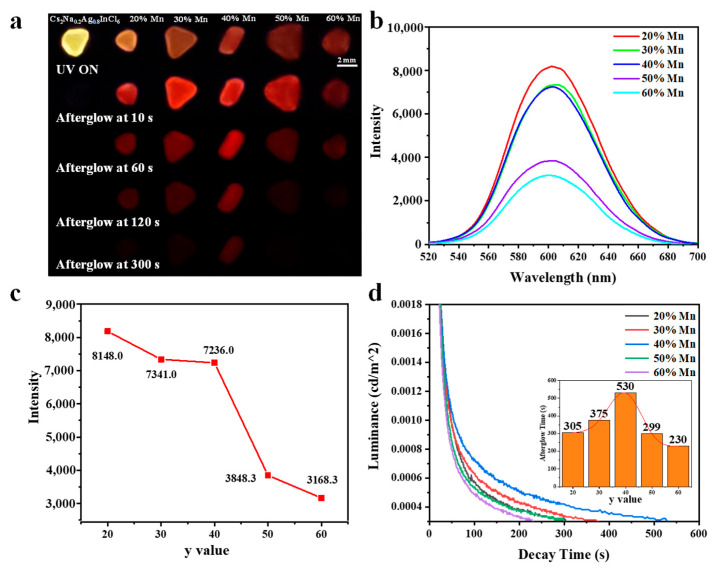
(**a**) Fluorescence and afterglow photos of the Cs_2_Na_0.2_Ag_0.8_InCl_6_:y%Mn crystals. (**b**) Afterglow spectra of the Cs_2_Na_0.2_Ag_0.8_InCl_6_:y%Mn. The samples were excited by 365 nm lamp for 10 min. (**c**) The afterglow intensity versus y value. (**d**) Afterglow decay traces of the Cs_2_Na_0.2_Ag_0.8_InCl_6_:y%Mn. Inset: the afterglow lifetime versus y value.

The actual Mn^2+^ concentrations of Cs_2_Na_0.2_Ag_0.8_InCl_6_:y%Mn (y = 0, 20, 30, 40, 50, 60) crystals are determined by X-ray photoelectron spectroscopy (Figures S1 and S2 and Table S1 in Supplementary Materials). The Cs_2_Na_0.2_Ag_0.8_InCl_6_:y%Mn were continuously irradiated by a 365 nm lamp for 10 min. Luminescence and afterglow photos of them are shown in Figure 1a. As expected, Cs_2_Na_0.2_Ag_0.8_InCl_6_ without Mn^2+^ doping does not exhibit any afterglow. As the Mn^2+^ concentration increases, the visible afterglow time first increases then decreases, reaching a maximum for nominal y = 40. The afterglow spectra captured after removing the excitation are shown in Figure 1b. All of the spectra mainly show an afterglow emission peak at ca. 600 nm owing to the Mn^2+ 4^T_1_→^6^A_1_ transition [19,20,21]. The transition of the d-orbital electrons associated with the Mn^2+^ ions is spin-forbidden [22]. Only when the Mn^2+^ ion is doped in a crystal is the transition allowed, due to the coupling with the host crystal’s field [23]. The finite hybridization with the host electronic states gives rise to an energy spread of Mn d states. The observation of broadband emissions at ca. 600 nm verifies the doping of Mn^2+^ into the metal halide crystal. As the Mn^2+^ concentration increases, the afterglow intensity decreases monotonically (Figure 1c). The afterglow decay traces are plotted in Figure 1d. With increasing Mn^2+^ doping, the afterglow time first increases from 305 s to 530 s, then decreases to 230 s (inset in Figure 1d). To obtain a more accurate understanding of the relationship between afterglow time and y value, we also prepared samples with y = 10, 35, and 45. Their afterglow decay traces are presented in Figure S3. These results manifest that both the afterglow intensity and time can be regulated by Mn^2+^ doping content.

**Figure 2:**
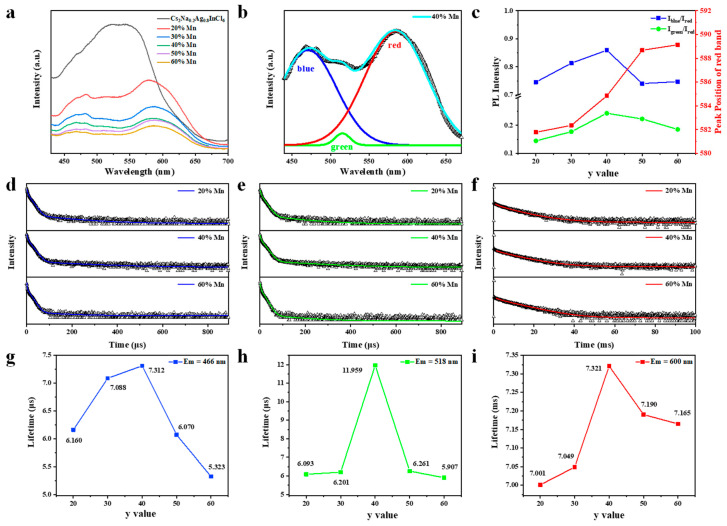
(**a**) Steady-state PL spectra of the Cs_2_Na_0.2_Ag_0.8_InCl_6_:y%Mn. (**b**) PL spectra of the Cs_2_Na_0.2_Ag_0.8_InCl_6_:y%Mn after normalizing the red band. Inset: Gaussian fitting of a selected PL spectrum of Cs_2_Na_0.2_Ag_0.8_InCl_6_:40% Mn. (**c**) Relative PL intensity and peak position of the red band versus Mn^2+^ doping concentration. (**d**–**f**) Time resolved PL spectra monitored at 466 nm (**d**), 518 nm (**e**), 590 nm (**f**). (**g**–**i**) The relationships between PL lifetimes and y value.

In order to gain more insight into the Mn^2+^ doping concentration dependent afterglow, the steady-state PL spectra and time-resolved PL decay curves were measured. As shown in Figure 2a, the overall PL intensity decreases monotonically with the increasing of Mn^2+^ doping concentration, which is consistent with variation of afterglow intensity, indicating the Mn^2+^ doping introduces nonradiative traps. The ultrabroad band involves disparate transitions obviously. To visually show the change of each peak, a single PL spectrum is Gaussian fitted into three sub-bands at about 466 nm, 518 nm, and 590 nm (Figure 2b and Figure S4), which are labeled as blue, green, and red peak, respectively. According to previous reports [21,24,25], the blue and green peaks with large full widths at half maximum (FWHM) and big Stokes-shift are attributed to self-trapped excitons (STE) caused by Jahn–Teller deformation of different metal chloride octahedra, i.e., [InCl_6_] and [AgCl_6_]. The red peak is due to the ^4^T_1_ to ^6^A_1_ transition of Mn^2+^ [19,20,21]. As shown in Figure 2c, the intensity of blue and green peak relative to red peak increase then decrease as the Mn^2+^ concentration increases. Apart from the change in PL intensity, the red peak undergoes a gradual shift from 581.8 nm to 589.1 nm with the increasing Mn^2+^ concentration.

The time-resolved PL spectra were measured at three emission wavelengths, which were fitted by tri-exponential decay functions (Figure 2d–f and S5–S7 and Tables S2–S4). The average PL lifetimes of blue and green emissions are about 5 to 10 micro-seconds, matching well with the typical lifetime of STEs [24,25,26]. The spin-forbidden Mn^2+^ d−d transition is partially relaxed due to the strong coupling with the host crystal [11,23,27], which usually occurs at time scale of milliseconds. The average PL lifetime of the red emission is about 7 ms, further confirming that the 590 nm-emission is related to the Mn^2+^ d–d transition [11,28]. As shown in Figure 2g–i, the average PL lifetimes of all three emissions first increase then decrease with the increasing of the Mn^2+^ concentration, which is consistent with the dependence of afterglow time on doping concentration.

**Figure 3:**
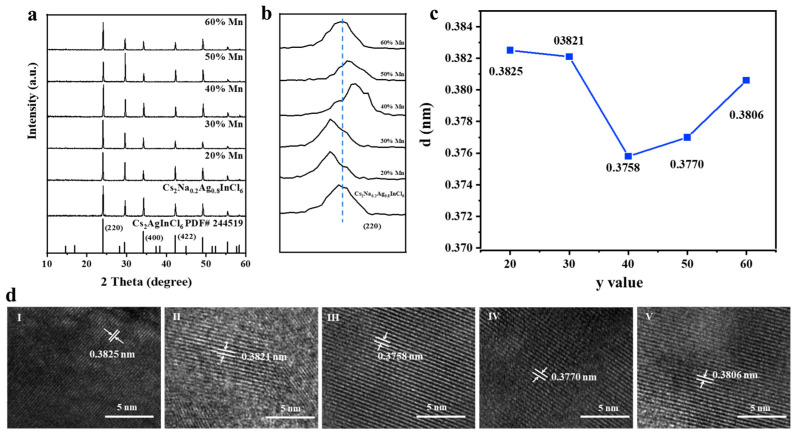
(**a**) The XRD patterns of the Cs_2_Na_0.2_Ag_0.8_InCl_6_:y%Mn and Cs_2_Na_0.2_Ag_0.8_InCl_6_. (**b**) The enlarged diffraction peak at 23.9°. The vertical dash line is used to highlight the peak shift. (**c**) The relationships between lattices spacing and y value according to HRTEM results. (**d**) HRTEM images of the Cs_2_Na_0.2_Ag_0.8_InCl_6_:y%Mn, I–V: the Mn^2+^ doping concentration in ascending order.

To understand the above observations in afterglow and PL, crystal structures of the Cs_2_Na_0.2_Ag_0.8_InCl_6_:y%Mn were investigated. As shown in Figure 3a, XRD patterns of the Cs_2_Na_0.2_Ag_0.8_InCl_6_:y%Mn show diffraction peaks similar to those of the undoped Cs_2_AgInCl_6_ with a space group Fm$\bar{3}$m, indicating the crystal is a face-centered cubic structure. It is worth noting that the characteristic peak corresponding to diffraction of (220) plane shows significant deviations with different amounts of Mn^2+^ doping (Figure 3b), suggesting different degrees of lattice distortion. This characteristic peak undergoes a shift to smaller angles first and then returns, implying the lattice of co-doped crystal first contracts and then expands with the increasing of the Mn^2+^ concentration. By analyzing the crystal structure, the Ag and In are found to be possible substitution sites [29,30]. The radius of Mn^2+^ (0.90 Å) is smaller than that of Ag^+^ (1.15 Å). The replacement of Ag^+^ by Mn^2+^ leads to the lattice shrinkage. Substitution of In^3+^ (0.8 Å) by Mn^2+^ (0.90 Å) leads to an expansion of the lattice. Excessive doping brings interstitial Mn^2+^, also leading to an expansion of the lattice. The variation of lattice spacing was further verified by HRTEM results (Figure 3c,d).

**Figure 4:**
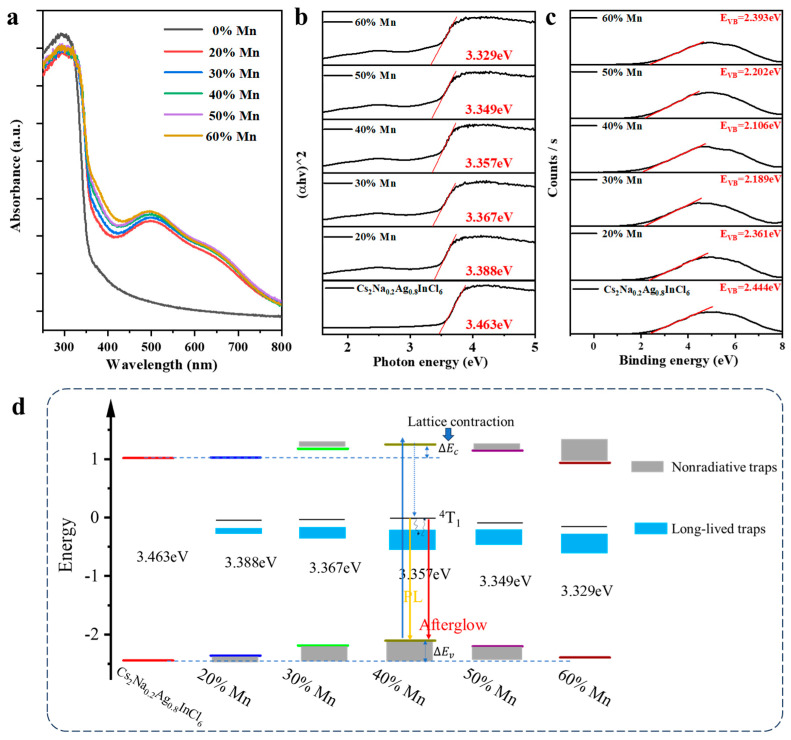
(**a**) UV–vis absorption spectra of the Cs_2_Na_0.2_Ag_0.8_InCl_6_:y%Mn. (**b**) Tauc plots of (αhν)^2^ versus hν. (**c**) VB XPS spectra of the Cs_2_Na_0.2_Ag_0.8_InCl_6_:y%Mn. (**d**) Scheme illustrating the band structures of the Cs_2_Na_0.2_Ag_0.8_InCl_6_:y%Mn.

The Mn^2+^ doping-induced lattice distortion leads to the change of band structure. The UV–vis diffuse reflectance spectra were measured to estimate the optical band gaps (Figure 4a). The absorbance at 497 nm caused by Mn^2+^ transition to monotonously increase with y% value (Figure S8). As well established previously, this type of metal halides is a direct bandgap semiconductor [31,32]. As shown in Figure 4b, the direct bandgaps of Cs_2_Na_0.2_Ag_0.8_InCl_6_:y% with y = 20, 30, 40, 50, 60 are calculated to be 3.463 eV, 3.388 eV, 3.367 eV, 3.357 eV, 3.349 eV, 3.329 eV, respectively, as calculated by the Tauc method [33]. The bandgap decreases with increasing Mn^2+^ doping (Figure S9), explaining the redshift of the red peak. To gain more insight into the band structures, the valence band positions were measured by ultraviolet photoelectron spectroscopy (UPS). As shown in Figure 4c, the valence band positions show a trend of first increasing and then decreasing, reaching a maximum at y = 40. According to the bandgap values and valence band positions, the band structures of Cs_2_Na_0.2_Ag_0.8_InCl_6_:y%Mn are schematically shown in Figure 4d.

Understanding the complex role of Mn^2+^ is the key to explaining the PL and afterglow. On the one hand, the Mn^2+^ doping leads to lattice deformation. The lattice contraction increases the bandgap while the lattice extension decreases the band gap. On the other hand, replacing monovalent Ag^+^ or trivalent In^3+^ with divalent Mn^2+^ necessarily disrupts local charge neutrality. This charge imbalance must be offset by compensatory defects such as vacancies or antisite defects. The Mn^2+^ doping, in particular excessive doping, introduces nonradiative traps (NTs) near the band edges, which reduce the bandgap. The synthetical factors result in monotonous narrowing of the bandgap. It is worth noting that the lattice distortion not only changes the bandgap but also affects the formation of STEs in the soft perovskite matrix. The appropriate lattice distortion is favorable to Jahn–Teller deformation and thus encourages STE formation [34,35]. However, excessive doping causes rapid nonradiative transition. Thereby, the relative PL intensity and lifetime corresponding to STE formation first increases, then decreases. Moreover, the Mn^2+^ doping introduces not only NTs but also LLTs that are responsible for the afterglow. The replacement of Ag^+^ with Mn^2+^ leads to the appearance of interstitial Ag^+^, which plays the role of LLTs. Under UV excitation, the interstitial Ag^+^ captures the photogenerated electrons, forming Ag, as demonstrated by the XPS results (Figure S10). In other words, the photogenerated electrons are temporally stored. After the removal of UV excitation, the residual holes recombine with the stored electrons, emitting the afterglow. At y = 40, the lattice shrinkage reaches its maximum, implying the highest concentration of interstitial Ag^+^, which agrees well with the longest afterglow time among the five samples. Excessive Mn^2+^ doping at interstitial sites does not bring about more LTTs, but rather introduces NTs. Thus, the afterglow time decreases when y exceeds 40.

We would like to point out that the PL and the afterglow are two different photophysical processes. For the PL process, the Mn^2+^ is excited by energy transfer from the Cs_2_Na_0.2_Ag_0.8_InCl_6_ without the participation of LLTs. By contrast, the afterglow is caused by the release of electrons trapped by of LLTs to Mn^2+^. The afterglow time essentially depends on the electron storage by LLTs, which lasts hundreds of seconds. However, they have an inherent connection. The relatively long PL lifetime of Mn^2+^ (ms scale) is crucial to the appearance of afterglow. If the radiative recombination is ns scale, the excited states return to ground state via radiative transition before the storage by LLTs. Thus, afterglow is absent. Afterglow is usually observed in Mn/rare earth ion doped crystals, since the radiative transitions of Mn/rare earth ions are relatively slow, allowing the storage of excited states before the radiative transition [2,3,4,5,6].

**Figure 5:**
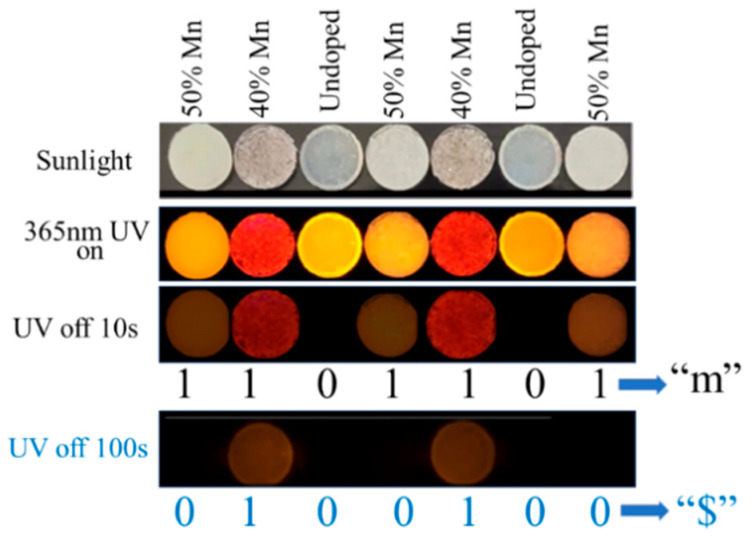
Applications of the Cs_2_Na_0.2_Ag_0.8_InCl_6_, Cs_2_Na_0.2_Ag_0.8_InCl_6_:40%Mn, and Cs_2_Na_0.2_Ag_0.8_InCl_6_:50%Mn in optical information storage and encryption.

Based on the afterglow time modulated by the Mn^2+^ doping concentration, we demonstrated an optical information storage and encryption application. Seven disks were fabricated by incorporating the Cs_2_Na_0.2_Ag_0.8_InCl_6_, Cs_2_Na_0.2_Ag_0.8_InCl_6_:40%Mn, and Cs_2_Na_0.2_Ag_0.8_InCl_6_:50%Mn into polydimethylsiloxane (PDMS). As shown in Figure 5, all of the disks showed similar colors under sunlight and similar orange-red fluorescence under 365 nm UV excitation. The information is hidden. After removing the UV excitation, the Cs_2_Na_0.2_Ag_0.8_InCl_6_ based disk becomes completely dark immediately, while other ones exhibit similar afterglow. By assigning the red disk as “1” and dark disks as “0”, the seven disks represent “1101101”, which are decoded to be “m” in the ASCII table. Furthermore, when the UV off lasts for 100 s, the afterglow of Cs_2_Na_0.2_Ag_0.8_InCl_6_:50%Mn based disks almost disappears. Consequently, the code turns into “0100100”, representing “$”. Supposing “$” is the only correct information, it is protected by the interference information “m”. Therefore, this dynamic strategy processes a high security for information encryption.

## 4. Conclusions

In summary, Cs_2_Na_0.2_Ag_0.8_InCl_6_:y%Mn crystals with afterglow emission at about 600 nm were synthesized by a solvothermal method. The afterglow mechanism is elucidated by establishing a correlation between crystal structure, band structure, and afterglow time. As the nominal y increased from 20 to 60, the afterglow time increased from 350 s to 530 s and then decreased to 230 s, reaching a maximum at y = 40. The steady-state PL spectra, time resolved PL, diffuse reflectance spectra, and UPS of the Cs_2_Na_0.2_Ag_0.8_InCl_6_:y%Mn crystals of similar size were measured and analyzed. On the one hand, Mn^2+^ doping leads to lattice deformation, which not only changes the bandgap but also affects the formation of STEs. On the other hand, Mn^2+^ doping introduces nonradiative traps near the band edges. Thus, both the overall PL intensity and the afterglow intensity monotonically decrease with increasing Mn^2+^ concentration. Furthermore, the replacement of Ag^+^ with Mn^2+^ leads to the appearance of interstitial Ag^+^, which temporally stores the photogenerated electrons by changing valence. However, excessive Mn^2+^ doping at interstitial sites does not bring about more interstitial Ag^+^ but rather introduces nonradiative traps. Thus, the afterglow time first increases then decreases as the Mn^2+^ doping concentration increases. We propose an optical information storage and encryption application based on the modulated afterglow time.

## Data Availability

The data presented in this study are available on request from the corresponding author.

## Supplementary Materials

The following supporting information can be downloaded at: https://www.mdpi.com/article/10.3390/nano15131002/s1, Figure S1. XPS survey spectra of the Cs_2_Na_0.2_Ag_0.8_InCl_6_:y%Mn. Figure S2. Cs 3d (a), Ag 3d (b), Na 1s (c), In 3d (d), Cl 2p (e), and Mn 2p (f) core level XPS spectra of Cs_2_Na_0.2_Ag_0.8_InCl_6_:40%Mn. Table S1. Actual Mn concentration measured by XPS. Figure S3. (a) Afterglow decay traces of Cs_2_Na_0.2_Ag_0.8_InCl_6_:y%Mn, y = 10, 35, 45. (b) The afterglow lifetime versus y value. Figure S4. Gaussian fittings of the PL spectra of Cs_2_Na_0.2_Ag_0.8_InCl_6_:y%Mn. Figure S5. Time resolved PL decay curves of the Cs_2_Na_0.2_Ag_0.8_InCl_6_:y%Mn monitored at 466 nm. Figure S6. Time resolved PL decay curves of the Cs_2_Na_0.2_Ag_0.8_InCl_6_:y%Mn monitored at 518 nm. Figure S7. Time resolved PL decay curves of the Cs_2_Na_0.2_Ag_0.8_InCl_6_:y%Mn monitored at 590 nm. Table S2. Fitting parameters for the PL lifetime curves monitored at 466 nm. Table S3. Fitting parameters for the PL lifetime curves monitored at 518 nm. Table S4. Fitting parameters for the PL lifetime curves monitored at 590 nm. Figure S8. Absorbance at 497 nm versus the y value. Figure S9. Band gaps determined by Tauc method versus y value. Error bars represent the standard deviations of repeated measurements. Figure S10. Ag 3d core level XPS spectra of Cs_2_Na_0.2_Ag_0.8_InCl_6_:40%Mn before and after UV illumination for 20 min.
