# Supplementary material for: Modulating the Afterglow Time of Mn2+ Doped Metal Halides and Applications in Advanced Optical Information Encryption
Source: Nanomaterials (Basel). 2025 Jun 28;15(13):1002. doi: 10.3390/nano15131002 (PMC12250681; doi:10.3390/nano15131002)
Supplement: Supplementary file 1 [file nanomaterials-15-01002-s001.zip › nanomaterials-3693305-supplementary.pdf]

Supplementary Material

Modulating the Afterglow Time of Mn^{2+} Doped Metal Halides and Applications in Advanced Optical Information Encryption

Yu-Lin Hu [1], Yi-Lin Zhu [1], Shi-Ying Gu [2], Jia-Qing Xu [3], Zhi-Xing Gan [4,*] and Chuan-Guo Shi [1,*]

[1] School of Chemistry and Chemical Engineering, Nantong University, Nantong 226019, China
[2] School of Public Health, Nantong University, Nantong 226019, China
[3] School of Intelligent Manufacturing and Electronic Engineering, Wenzhou University of Technology, Wenzhou 325035, China
[4] School of Computer and Electronic Information/School of Artificial Intelligence, Nanjing Normal University, Nanjing 210023, China

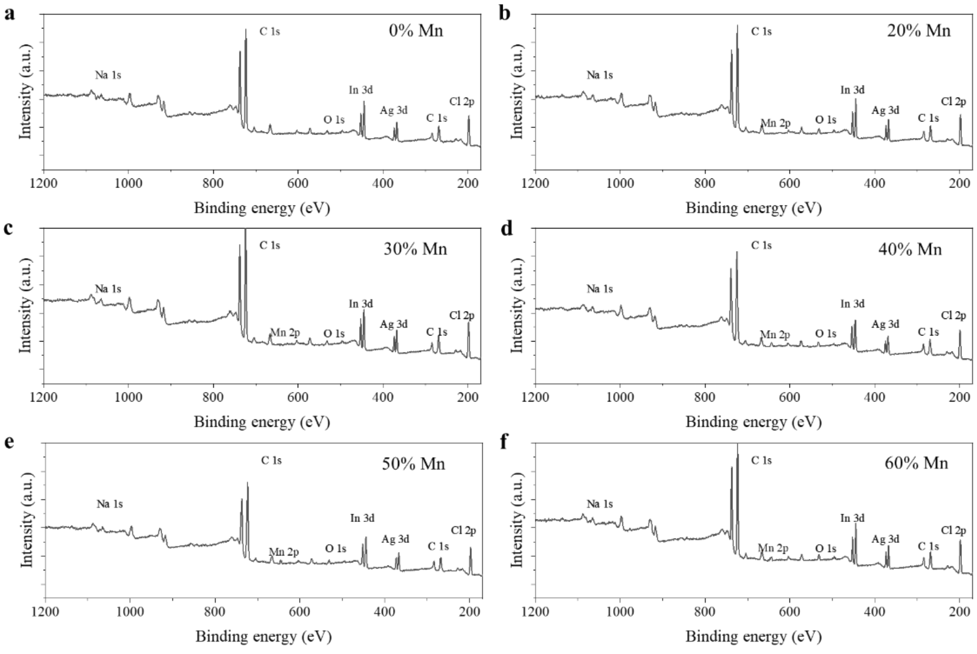

Figure S1. XPS survey spectra of the $Cs_2Na_{0.2}Ag_{0.8}InCl_6$:y%Mn.

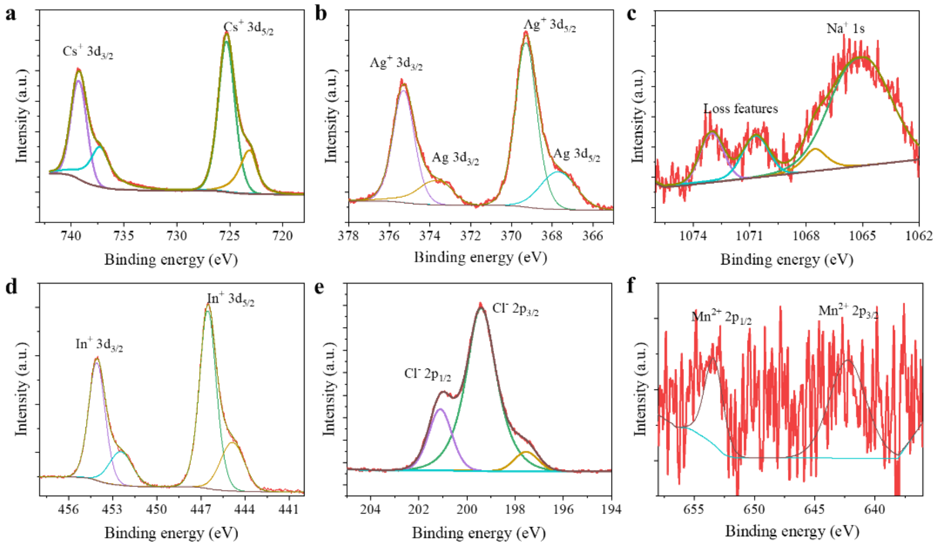

Figure S2. Cs 3d (a), Ag 3d (b), Na 1s (c), In 3d (d), Cl 2p (e), and Mn 2p (f) core level XPS spectra of $Cs_2Na_{0.2}Ag_{0.8}InCl_6$:40%Mn.

Table S1. Actual Mn concentration measured by XPS.

Nominal y	Actual Mn concentration wt%
20	1.12
30	1.96
40	2.30
50	2.71
60	3.02

Please note 20-60 is nominal y value in $Cs_2Na_{0.2}Ag_{0.8}InCl6$:y%Mn rather than nominal Mn doping concentration in wt%. These two quantities are different. However, there is a relationship between these two quantities.

Mn concentration

$$(wt\%)=\frac{y\%\times 54.94}{2\times 132.91+0.2\times 22.99+0.8\times 107.87+1\times 114.82+6\times 212.7+y\%\times 54.94}$$

Supposing y=20, Mn concentration in wt% is calculated to be 1.6%.

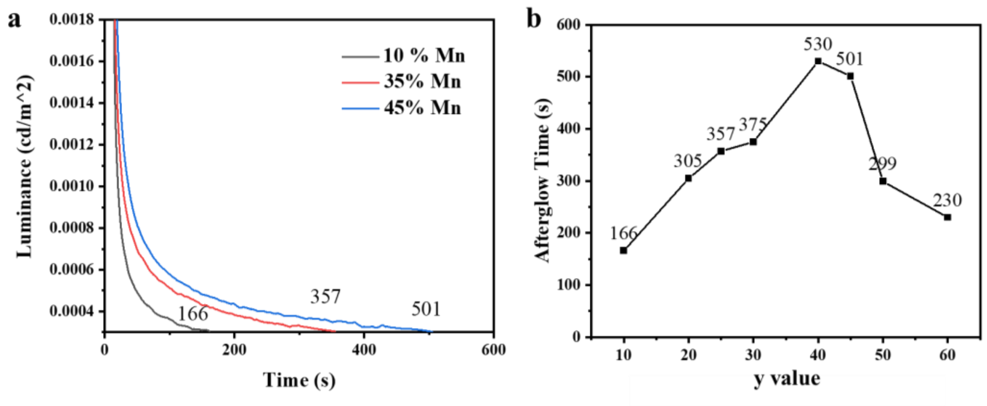

Figure S3. (a) Afterglow decay traces of $Cs_2Na_{0.2}Ag_{0.8}InCl_6$:y%Mn, y=10, 35, 45. (b) The afterglow lifetime versus y value.

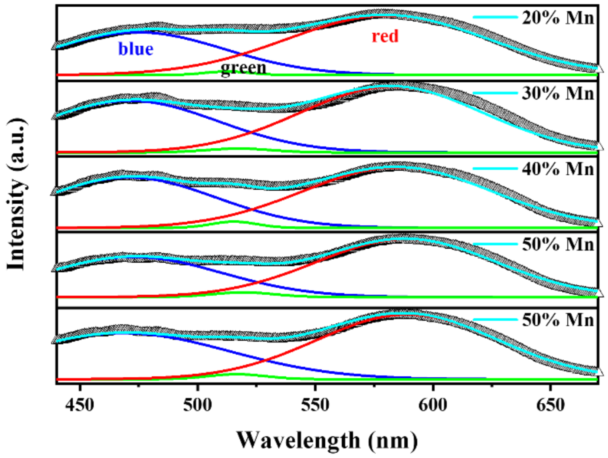

Figure S4. Gaussian fittings of the PL spectra of $Cs_2Na_{0.2}Ag_{0.8}InCl_6$:y%Mn.

The time-resolved PL spectra were monitored at emission wavelengths of 466 nm, 518 nm, 600 nm, which were fitted by tri-exponential decay functions. And the average PL lifetimes were calculated by equation:

$$\tau_A = \frac{A_1{\tau_1}^2 + A_2{\tau_2}^2 + A_3{\tau_3}^2}{A_1\tau_1 + A_2\tau_2 + A_3\tau_3} \tag{1}$$

where τ_1, τ_2 and τ_3 are the PL lifetimes and A_1, A_2 and A_3 are the corresponding amplitudes obtained by tri-exponential fittings. The fitting details are listed in Table S1-S3.

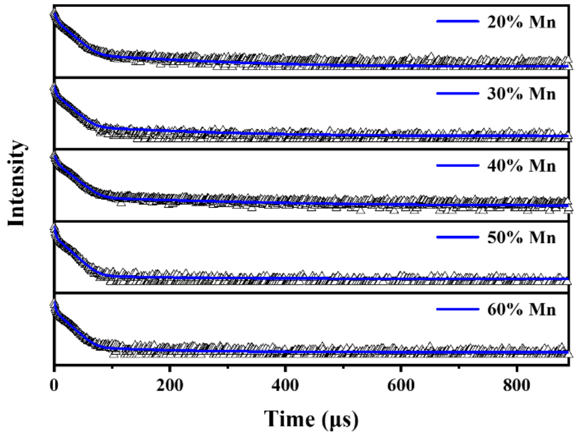

Figure S5. Time resolved PL decay curves of the $Cs_2Na_{0.2}Ag_{0.8}InCl_6$:y%Mn monitored at 466 nm.

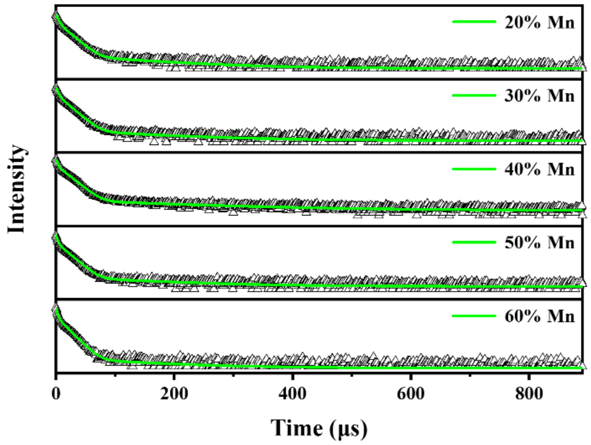

Figure S6. Time resolved PL decay curves of the $Cs_2Na_{0.2}Ag_{0.8}InCl_6$:y%Mn monitored at 518 nm.

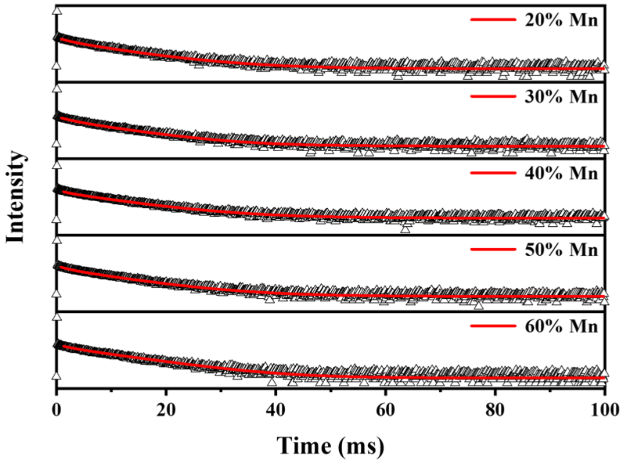

Figure S7. Time resolved PL decay curves of the $Cs_2Na_{0.2}Ag_{0.8}InCl_6$:y%Mn monitored at 590 nm.

Table S2. Fitting parameters for the PL lifetime curves monitored at 466 nm.

Nominal y	A_1	T_1 (μs)	A_2	T_2 (μs)	A_3	T_3 (μs)	T_a (μs)	R^2
20	178529.1	2.243	45057.09	9.189	21.864	123.0	6.160	0.99881
30	108455.1	1.48	28905.07	9.215	18.837	136.7	7.088	0.98864
40	332629.3	1.318	66534.75	9.756	26.866	200.6	7.312	0.96339
50	58756.3	1.339	9827.77	9.566	2.07	148.2	6.070	0.98972
60	120009.4	1.344	19367.08	8.702	5.758	114.8	5.323	0.96586

Table S3. Fitting parameters for the PL lifetime curves monitored at 518 nm.

Nominal y	A_1	T_1 (μs)	A_2	T_2 (μs)	A_3	T_3 (μs)	T_a (μs)	R^2
20	116168.8	1.685	27996.13	8.839	13.239	120.8	6.093	0.98871
30	134607.6	2.478	36412.36	9.285	14.012	122.3	6.201	0.99551
40	20075.52	3.051	47731.91	10.551	21.888	245.5	11.959	0.95216
50	116168.8	1.985	27996.13	9.239	13.238	130.8	6.261	0.98896
60	58396.62	1.809	12344.94	9.135	4.538	126.2	5.907	0.99836

Table S4. Fitting parameters for the PL lifetime curves monitored at 590 nm.

Nominal y	A_1	T_1 (μs)	A_2	T_2 (μs)	A_3	T_3 (μs)	T_a (μs)	R^2
20	160.621	1.467	346.952	5.342	144.127	10.03	7.001	0.96678
30	266.03	2.344	471.95	7.406	13.216	15.35	7.049	0.97152
40	313.158	1.236	674.643	5.746	254.596	10.49	7.321	0.99286
50	238.07	1.501	521.677	6.257	113.803	11.18	7.190	0.97700
60	227.037	2.106	491.665	7.563	11.503	13.31	7.165	0.97897

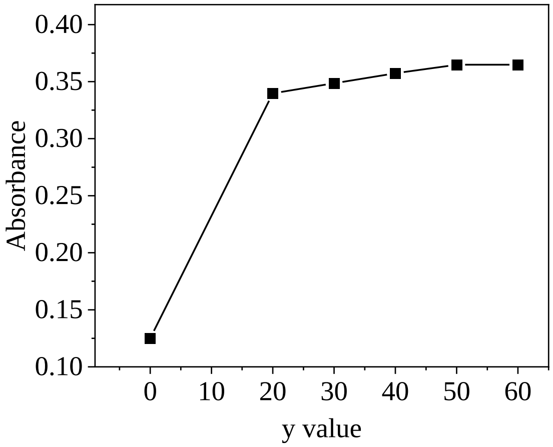

Figure S8. Absorbance at 497 nm versus the y value.

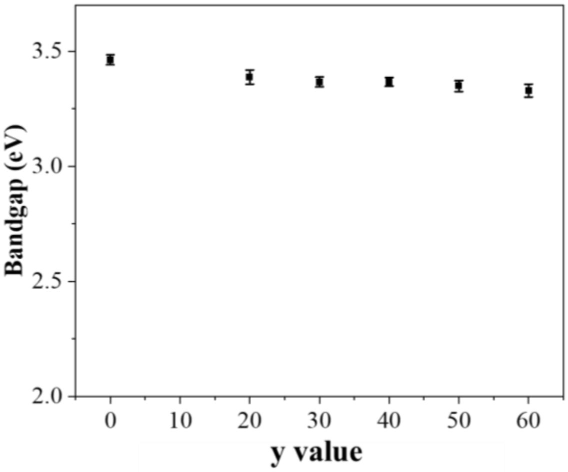

Figure S9. Band gaps determined by Tauc method versus y value. Error bars represent the standard deviations of repeated measurements.

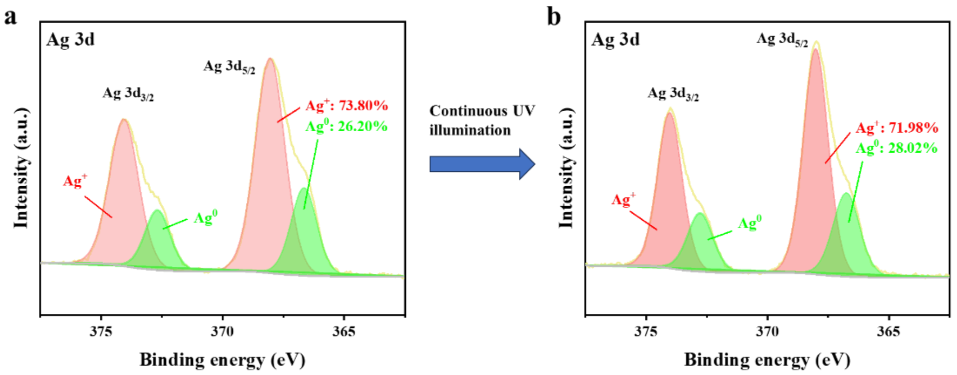

Figure S10. Ag 3d core level XPS spectra of $Cs_2Na_{0.2}Ag_{0.8}InCl_6$:40%Mn before and after UV illumination for 20 min.
